# Response to treatment and low serum vitamin D levels in North Indian patients with treatment-naive category I and multi-drug resistant pulmonary tuberculosis

**DOI:** 10.1080/07853890.2024.2407066

**Published:** 2024-09-23

**Authors:** Jaishriram Rathored, Surendra Kumar Sharma, Jayant Nagesh Banavaliker, V. Sreenivas, Abhay Krishna Srivastava

**Affiliations:** a Department of Medicine, All India Institute of Medical Sciences, New Delhi, India; b Department of ‘School of Allied Health Sciences’, Datta Meghe Institute of Higher Education and Research, Wardha, Maharashtra, India; c Department of Tuberculosis and Respiratory Diseases, Rajan Babu Institute of Pulmonary Medicine and Tuberculosis (RBIPMT), New Delhi, India; d Department of Biostatistics, All India Institute of Medical Sciences, New Delhi, India; e Department of Laboratory Medicine, All India Institute of Medical Sciences, New Delhi, India

**Keywords:** Multi-drug resistant tuberculosis, vitamin D, smear and culture conversion, Cat I pulmonary tuberculosis, treatment response

## Abstract

**Background:**

Tuberculosis (TB) is a bacterial infection that usually affects the lungs, although it can also affect other parts of the body. Vitamin D deficiency and response to treatment have been demonstrated in patients with active TB in several studies, but not in MDR-TB patients, which is a new observation in the present study.

**Objective:**

To study the time to initial sputum culture conversion and to associate baseline vitamin D levels and response to treatment in patients with PTB Cat I and MDR-TB.

**Methods:**

A total of 897 North Indian participants were recruited and divided into three groups: treatment-naïve PTB Cat I, MDR-TB, and healthy controls. Serum biochemistry, including 25-hydroxyvitamin D and calcium, was measured in all participants with PTB, Cat I, and MDR-TB.

**Results:**

PTB Cat I patients had high bacillary load grading at baseline compared to 2^nd^ month followed by 6^th^ month of treatment. More severe chest radiographic features, such as cavitation and the presence of bilateral disease at baseline. Mean sputum smear conversion times were 0.95 ± 0.7 months and culture conversion to negative occurred at a mean time of 0.8 ± 0.7 in PTB Cat I patients compared to MDR-TB patients on average sputum smear and time of 2.4 ± 3 months. Significantly lower mean serum 25-hyroxyvitamin D concentration was found in the 6^th^ month than in the 2^nd^ month and baseline in PTB Cat I.

**Conclusion:**

Low serum vitamin D deficiency was observed in both groups during treatment and is one of the important factors responsible for susceptibility to TB in both groups; however, its significance is uncertain. Patients with continuous positive sputum for multidrug-resistant tuberculosis (MDR-TB) had a worse prognosis than those with sputum bacteriology conversion. Two months into a treatment regimen, sputum smear conversions may be a useful indicator of an MDR-TB patient’s prognosis.

## Introduction

Tuberculosis (TB) is a potentially deadly infectious disease that commonly affects the lungs. It is a multifactorial disease in which the environment interacts with host-related factors to contribute to the overall clinical spectrum [1]. It is a significant cause of morbidity and mortality and causes approximately 1.3 million deaths annually among HIV-negative TB cases of TB and approximately 0.4 million were in people are co-infected with the human immunodeficiency virus (HIV) [2]. The patients’ lung tissue is destroyed by the infection, which makes them cough up the bacteria. The germs then travel through the air and can be breathed by other people. In patients with TB who are resistant to multiple drugs, isoniazid and rifampicin are considered the most potent anti-TB medications [3]. It has also been observed that due to low adherence to anti-tuberculosis treatment (ATT) and malnutrition, drug-susceptible TB may be converted to MDR-TB, often the most clinically severe and deadly form of the disease [4].

Studies have suggested that vitamin D acts as a potent immunomodulator of innate immune responses [5–7] by acting as a co-factor for the induction of antimycobacterial activity [8]. 1,25-Dihydroxyvitamin D3 [1,25-(OH)2D3] is the main regulator of plasma calcium and phosphorus levels in tissues [9]. In the liver, vitamin D3 undergoes two-step hydroxylation and then in the kidney to form the active metabolite 1,25-(OH)2D3. Serum ionized calcium (SiCa) is the biologically active fraction of serum calcium, and its concentration in serum varies within very narrow limits (1.15-1.35 mmol/l). Calcium homeostasis is tightly regulated by parathyroid hormone (PTH), 1, 25-(OH)_2_D, and calcitonin in the kidney, bone, and intestine. In the kidney, PTH induces a second hydroxylation step to form active vitamin D [10]. PTH is the main hormone regulating 1, 25-(OH)_2_D production *via* a negative feedback mechanism. PTH together with 1, 25-(OH)_2_ D, maintains serum calcium homeostasis by increasing bone calcium resorption, calcium reabsorption, and phosphate excretion in the kidneys, as well as intestinal calcium and phosphate absorption [11].

It is known that the principal source of vitamin D **is** sunlight, and that plasma concentrations of vitamin D have striking seasonal variation, with peak levels after summer and the lowest levels in spring. The occurrence of TB has been reported to be related to seasonal variations in vitamin D status in northern India [12,13]. Serum vitamin D level is the best indicator of overall vitamin D status because it reflects the total vitamin D from dietary intake and sunlight exposure, as well as the conversion of vitamin D from adipose stores in the liver [14].

A lack of vitamin D may maintain the chance of reactivation [15]. Numerous studies have linked vitamin D deficiency to TB, indicating that this deficiency may alter immune function that confers resistance against *Mycobacterium tuberculosis* [15–17]. Reduced levels of vitamin D in the blood may increase the chance of TB reactivation [15].

The link between vitamin D and TB has been increasing in passionate studies. Several studies have reported vitamin D deficiency has been established in patients with active TB [12,18–21]. Studies from diverse parts of the country revealed that 90% of the apparently healthy subjects in Delhi were classified either as vitamin D insufficient or deficient using serum 25(OH)D cut-off levels of 32 ng/ml and 20 ng/ml respectively [22,23]. Vitamin D deficiency (25-hydroxycholecalciferol) has long been implicated in TB [24]. Serum levels of vitamin D in patients with TB are lower than those in healthy controls [25,26]. Long-term TB treatment occasionally lowers serum vitamin D levels as well [25]. Before effective anti-tubercular therapy was developed, patients with TB were advised to receive treatment and recover at the sanatorium, which had plenty of sunshine. This study examined the differences in intact parathyroid hormone (iPTH), calcium (ionized and total), and vitamin D levels in pulmonary TB patients at baseline, two months, and six months. Sputum smears and cultures were done on MDR-TB patients once a month, or until the cases were either turned negative or the patients were released from the ward.

This study aimed to observe the conversion of sputum cultures that were positive to negative after treatment in PTB Cat I and MDR-TB and to identify any link between serum vitamin D levels and the correlation of biochemical parameters associated with quicker conversion.

## Patients/material and methods

### Study design and populations

In this cross-sectional study, 692 patients and 205 controls were recruited at baseline. Out of 354 MDR-TB patients admitted at the Rajan Babu Institute of Pulmonary Tuberculosis (RBIPMT) hospital, only 236 were enrolled; of the 338 newly diagnosed sputum-positive pulmonary TB Cat I patients at the All-India Institute of Medical Sciences (AIIMS) hospital, New Delhi only 60 patients aged between 18-60 years were recruited between July 2006 and January 2011. All MDR-TB patients in this study received treatment with 2^nd^ line drugs or a DOTS-plus regimen, and the PTB Cat I group contained newly diagnosed sputum smear- and culture-positive pulmonary cases, with baseline susceptibility to all first-line anti-tuberculosis drugs.

Consecutive patients were enrolled after strict exclusion criteria were applied using a structured questionnaire, namely, TB patients in category II or MDR-TB treatment as per the Indian Revised National Tuberculosis Control Programme (RNTCP) guidelines [27]. The presence of secondary immunodeficiency, for example, corticosteroid or other immunosuppressant drug use, diabetes mellitus, malignancy, co-infection with HIV, hepatitis B or hepatitis C virus, extrapulmonary TB in the absence of pulmonary involvement, concurrent cytotoxic chemotherapy, pregnancy or lactation, current or recent (<1year) use of vitamin D and/or calcium supplements; and patients with known seizure disorder. Only patients with sputum smears and positive culture results were enrolled in the study. All patients were clinically examined, their sputum smear and culture reports were recorded, the times between the start of treatment and negative sputum cultures were calculated, and characteristics associated with sputum culture conversion were examined. Sputum smears and cultures were done on MDR-TB patients once a month or until the patients had been discharged from the ward or their results were converted to negativity. Recruitment was done for healthy subjects who shared the same socioeconomic background as the northern Indian population as a whole. Written informed consent was obtained from all the patients. The study protocol was approved by the Institutional Ethics Committee of AIIMS (Ref no.: A-08/5.5.2008).

Sample size was calculated by considering mean difference value of MDR-TB, PTB cat I and control group over the parameter of 25 (OH)D found as 5.9 as per Rathored J et al. [18] and estimated standard deviation of 20 with desired power of 0.80 and alpha error of 0.05 minimum sample size required as 181 per group.

### Assessment

Patients were clinically examined, and a posteroanterior plain chest radiograph was obtained to characterize radiographic severity [28]. Demographic data, full medical history, including previous history of TB and/or previous contact with individuals with TB, and drug and alcohol history were recorded. At least two baseline sputum samples, one of which was an early morning sample, were obtained from each patient according to the RNTCP guidelines [27]. The specimens were placed on ice packs and transported to the New Delhi TB Center laboratory in New Delhi (accredited by RNTCP as an intermediate reference laboratory) within 24 h of collection [27]. All Samples were examined for the presence of mycobacteria using a standard Ziehl-Neelsen staining technique, and bacillary load was graded using internationally recognized WHO guidelines [29]. *Mycobacterium tuberculosis* culture was performed on the Lowenstein-Jensen slopes. Conventional drug susceptibility testing using the proportion method was performed on all samples, using the definitions explained above for classification as drug susceptible or MDR-TB.

### Treatment and monitoring

According to the World Health Organization’s recommendations [2] all patients received anti-tuberculosis chemotherapy in compliance with the RNTCP guidelines for directly observed therapy [27]. Following a two-month initial phase of weekly rifampicin, isoniazid, pyrazinamide, and ethambutol, patients with a new diagnosis of TB with full drug susceptibility were placed on a six-month rifampcin-based regimen, which was followed by weekly rifampicin and isoniazid in the continuation phase [27].

Patients were followed-up every week during the initiation phase and every two weeks during the continuation phase. Clinical assessment, sputum smear, and culture were conducted on a minimum of two samples at each review, one of which was generated early in the morning. Patients who were susceptible to drugs underwent weekly sputum smear microscopy and biweekly culture procedures until negative results were achieved. Sputum smears, cultures, and chest radiographs were subsequently reviewed three times during the course of the treatment: before it began, at the end of the second month, and in the sixth and final month. Medical social workers were assigned to provide support in addition to the close monitoring of treatment adherence and response by medical officers, nursing staff, and treatment supervisors.

### Drug-Susceptibility testing and treatment

At the beginning of the enrollment period, the standard drug susceptibility test panel comprised of isoniazid, rifampin, ethambutol, pyrazinamide, and streptomycin. Drug susceptibility testing was conducted at the New Delhi TB Centre. From AIIMS outpatient care, hospital-bound patients were transferred to RBIPMT, New Delhi. Every month, sputum samples were gathered for local laboratories to use for smear microscopy and culture.

### Serum vitamin D, iPTH and calcium levels

After overnight fasting without venostasis, blood samples for vitamin D and iPTH were drawn from all patients. The serum was separated in a refrigerated centrifuge at 2500 × *g* for 5 min at 4 °C and stored at −80 °C in multiple aliquots until analysis. Serum 25-hydroxyvitamin D concentrations were estimated using the Diasorin^®^ 25-hydroxyvitamin D RIA (normal range: 9–37.6 ng/mL), which involves a two-step procedure. The first step involves the extraction of 25-hydroxyvitamin D from serum with acetonitrile, followed by processing according to the manufacturer’s instructions. Serum iPTH levels were measured using radioimmunoassay (RIA; Diasorin^®^, Stillwater, MN; normal range: 13–54 pg/mL; intra-assay and inter-assay CVs: 6% and 9%, respectively).

A Carelyte Electrolyte Analyzer (Carewell Biotech Pvt Ltd, New Delhi, India) was used to measure serum ionized calcium using an ion selective electrode method (PH-range 6.5-8.5 nmol/L). Resolution: 0.01%, CV: less than 1%. Following collection, serum samples were kept at 4 °C and separated from cells in less than an hour. Special care was taken to ensure that no air bubbles that could destroy ionized calcium were included in the serum samples. Similarly, the o-cresolphthalein complexone method was used to estimate serum total calcium using a Hitachi Modular (Roche Diagnostics GmbH, Mannheim, Germany). Radioimmunoassay (RIA; Diasorin^®^, Stillwater, MN, USA) was used to measure the levels of iPTH in serum (reference interval: 13–54 pg/mL; intra-assay and inter-assay coefficients of variation: 4% and 8%, respectively).

### Statistical analysis

Quantitative variables such as age and BMI were compared among the three groups of subjects using one-way analysis of variance (ANOVA) followed by Bonferroni correction for multiple comparisons. Spearman’s rho correlation coefficient test was used for correlation analysis Biochemical analysis is based on the XtGee generalized estimating equation. A two-sided p-value of less than 0.05 was considered statistically significant. Data are presented as mean ± standard deviation (SD). Additionally, median (IQR) values for the times it takes for sputum smear conversion and the culture’s conversion to negative are also provided. All analyses were performed using Stata version 11.0 (Stata Corporation, College Station, TX).

## Results

### Severity of disease

Table 1 presents a comparison of the clinical and demographic features of patients with PTB cat I and MDR-TB. Table 2 displays the disease severity as determined by sputum smear, culture, and chest radiography. In comparison to the PTB Cat I, the MDR-TB group had a lower bacillary load (*p* < 0.001), more severe chest radiographic features (*p* = 0.02), including multiple cavitations and far advanced disease, and the presence of bilateral disease (*p* < 0.001) (Table 2). MDR-TB (*in vitro* demonstration of *Mycobacterium tuberculosis* resistance to isoniazid and rifampicin) diagnosis required laboratory testing; 15% of PTB Cat I cases were culture-negative (Table 2). PTB cat I patients had more severe chest radiographic features, such as cavitations and the presence of bilateral disease at baseline, as well as higher bacillary load grading at baseline compared to the second month and low or negative following the sixth month of treatment (Table 3).

**Table 1.** Baseline clinical characteristic of patients with MDR-TB, PTB cat I and healthy controls.

	MDR-TB	PTB Cat I		
Characteristics	(*n* = 354)	(*n* = 338)	Healthy controls (*n* = 205)	*p*-value*
Age	27.5 ± 10.4	27.4 ± 9.6	29.0 ± 8.9	0.13
Sex M: F	228:126	243:95	140:65	0.11
BMI (kg/m^2^)	15.8 ± 2.5	17.5 ± 2.5	23.4 ± 3.0	<0.001
BCG Scar				
Positive	42 %	79%	59%	<0.001
Negative	58%	21%	41%	
Smoking				
Yes	51%	27%	22%	<0.001
No	49%	73%	78%	
Alcohol^†^				
Regular	44%	42 %	9%	<0.001
Occasional	7%	0%	0%	
No	49%	58%	91%	

Plus–minus values are means ± SD; *p-value less than 0.05 was considered statistically significant; Regular= (250 ml) 2-3 times a week, occasional= 1-2 times a month; all recruited patients and controls were HIV negative; MDR-TB: multi-drug resistant tuberculosis; PTB Cat I: pulmonary TB Category I; M: male; F: female; BMI: body mass index; BCG: Bacillus Calmette-Guérin.

**Table 2.** Baseline sputum (smear and culture) and radiographic severity of patients with MDR-TB and PTB cat I.

	MDR-TB patients	PTB Cat I patients	
	(*n* = 354), n (%)	(*n* = 338), n (%)	*p*-value*
^**^Bacillary load			
3+	87 (25)	145 (43)	
2+	102 (29)	75 (22)	<0.001
1+	161 (45)	86 (25)	
Scanty	04 (01)	32 (10)	
AFB Culture status			
Positive	354 (100)	286 (85)	<0.001
Negative	0 (00)	52 (15)	
^†^Radiographic severity			
Unilateral	24 (7)	120 (36)	<0.001
Bilateral	330 (93)	218 (64)	
Minimal	11 (3)	31 (09)	
Moderately advanced	94 (27)	226 (67)	<0.001
Far advanced	249 (70)	81 (24)	
Cavity			
Yes	314 (89)	280 (83)	0.02
No	40 (11)	58 (17)	
No. of cavity			
1	66 (19)	111 (34)	
2	62 (17)	61 (18)	–
3	48 (14)	31 (09)	
Multiple	138 (39)	77 (22)	

*p < 0.05, considered statistically significant; MDR-TB: multi-drug resistant tuberculosis; PTB Cat I: pulmonary TB Category I; AFB: acid-fast bacilli.

**Bacillary load grading of acid-fast bacilli counts on Ziehl-Neelsen stained sputum slides as per.

WHO grading guidelines [28].

†Chest radiograph severity grading was performed by consensus of two investigators as per the.

National Tuberculosis Association of the USA’s guidelines [29].

**Table 3.** Clinical characteristics of PTB Cat-I patients at three time points.

	0 day	2nd month	6th month
Characteristics	(*n* = 60)	(*n* = 60)	(*n* = 60)
^*^Bacillary load	48%	0%	0%
3+	14%	0%	0%
2+	30%	7%	0%
1+	8%	5%	0%
Scanty /Negative	0%	88%	100%
^†^X-ray severity			
Mild	12%	35 %	37%
Moderate	67%	65%	63%
Far advanced	21%	0%	0%
Cavitary	80%	55%	5%
Non-cavitary	20%	45%	95%

*Bacillary load grading of acid-fast bacilli counts on Ziehl-Neelsen stained sputum slides as per.

WHO grading guidelines [28].

†Chest radiograph severity grading was performed by consensus of two investigators as per the.

National Tuberculosis Association of the USA’s guidelines [29].

Sputum smear and culture conversion data were available for 236 patients in the MDR-TB group and 60 patients with PTB Cat I (Tables 4 and 5). In the MDR-TB group, the mean sputum smear conversion times were 2.46 ± 1.41 and the median (IQR) was 2 (1) months; in the PTB Cat I patients, the mean was 0.95 ± 0.7 and the median (IQR) was 1 (1) month (Table 4). The mean duration for culture conversion from positive to negative was 2.64 ± 1.36 months for MDR-TB patients, and 0.8 ± 0.7 months for PTB Cat I patients, with a median (IQR) of 1 (1) month (Table 5). However, 354 patients with MDR-TB had positive cultures when starting treatment. Of these, 220 patients had cultures that converted to negative (on average within 2.4 months in sputum smear and 3 months in sputum culture), 31 patients died, and 40 patients did not convert to negativity. Patients who had previous treatment for MDR-TB, high bacterial load in the initial culture, X-ray abnormalities in both lungs with multiple cavities, and/or TB bacteria that were resistant to a larger number of drugs, took the longest to undergo conversion. Treatment outcomes were worse in patients who did not have negative sputum cultures within 3 months than in those who converted within 3 months.

**Table 4.** Time to sputum smear conversion in MDR-TB and PTB cat I patients during treatment.

	MDR-TB patients (*n* = 236)		PTB Cat I patients (*n* = 60)	
Sputum smear conversion (in months)	N (%)	Cumulative frequency (%)	N (%)	Cumulative frequency (%)
0	0	0	0	0
1	47(20.6)	20.6	43 (71.7)	71.7
2	79 (34.6)	55.2	13 (21.7)	93.4
3	52 (22.8)	78	4 (6.6)	100
4	27 (11.8)	89.8	–	–
5	11 (4.8)	94.6	–	–
6	2 (0.9)	95.5	–	–
7	–	–	–	–
8	1 (0.4)	95.9	–	–
9	2 (0.9)	96.8	–	–
No conversion	7 (3.2)	100	0	0
Sputum smear time conversion*	2.46 ± 1.41	–	0.95 ± 0.7	–
	Median (IQR)		Median (IQR)	
	2(1)		1(1)	

*Plus-minus values are means ± SD (in months), *p* < 0.001; MDR-TB: multi-drug resistance tuberculosis; PTB Cat I: pulmonary TB Category I; The numerical 0-9 represent sputum smear conversion in months; IQR = Inter Quartile Range.

**Table 5.** Time to sputum culture conversion in MDR-TB and PTB cat I patients during treatment.

	MDR-TB patients (*n* = 236)		PTB Cat I patients (*n* = 60)	
Sputum culture conversion (in months)	n (%)	Cumulative frequency (%)	n (%)	Cumulative frequency (%)
0	0 (0)	0	15 (25)	25
1	7 (3)	3	33 (55)	80
2	88 (37.3)	40.3	09 (15)	95
3	78 (33.1)	73.4	2 (3.3)	98.3
4	13 (5.5)	78.9	1 (1.7)	100
5	26 (11)	89.9	–	–
6	2 (0.8)	90.7	–	–
7	2 (0.8)	91.5	–	–
No conversion	20 (8.5)	100	0	0
Sputum culture time conversion*	2.64 ± 1.36		0.8 ± 0.7	
	Median (IQR) = 3 (1)		Median (IQR) = 1(1)	

*Plus-minus values are means ± SD (in months), *p* < 0.001; MDR-TB: multi-drug resistance TB; PTB Cat I: pulmonary TB Category I; The numerical 0-9 represent sputum smear conversion in months; IQR = Inter Quartile Range.

Details of the various biochemical parameters between the three time points of category I pulmonary TB are provided in Table 6. Significantly lower mean serum vitamin D levels D concentration was found in 6^th^ month as compared to the 2^nd^ month then baseline (9.8 ± 6.0 ng/mL; 10.9 ± 8.8 ng/mL; 11.6 ± 8.4 ng/mL; *p* = 0.08), which inversely correlated with mean serum iPTH concentration (39.8 ± 18.7 pg/mL; 33.2 ± 21.6 pg/mL; 29.4 ± 16.3 pg/mL; *p* < 0.001). Similarly, significantly lower mean serum ionised calcium (3.0 ± 0.9 mg/dL; 3.2 ± 0.6 mg/dL; 4.5 ± 0.3 mg/dL; *p* < 0.001) and total calcium concentrations (6.0 ± 1.9 mg/dL; 6.4 ± 1.1 mg/dL; 9.0 ± 0.5 mg/dL; *p* < 0.001) were found in baseline then 2^nd^ month and 6^th^ month respectively. Mean sputum smear conversion times were 0.95 ± 0.7 months and culture conversion to negative occurred at a mean time of 0.8 ± 0.7 in PTB Cat I patients but sputum smear and culture conversion time for PTB cat I showed no significant relationship to serum vitamin D levels (Table 6).

**Table 6.** Biochemical parameters of PTB Cat-I patients at three time points.

	0 day	2 months	6 months	
	Mean ± SD	Mean ± SD	Mean ± SD	
Biochemical parameters	(*n* = 60)	(*n* = 60)	(*n* = 60)	p* value
Serum total protein (g/dL)	8.0 ± 0.9	7.6 ± 1.2	8.0 ± 0.9	0.99
Serum albumin (g/dL)	3.9 ± 0.8	3.6 ± 0.9	4.0 ± 0.9	0.62
Serum globulin (g/dL)	4.6 ± 0.7	4.0 ± 0.8	3.9 ± 0.9	0.62
Serum ionized calcium (mg/dL	3.2 ± 0.6	3.3 ± 0.7	3.5 ± 0.7	<0.001
Serum calcium (mg/dL)	6.4 ± 1.1	6.7 ± 1.3	6.8 ± 1.4	0.12
Corrected serum calcium (mg/dL)	9.4 ± 1.6	9.0 ± 2.3	9.4 ± 2.6	0.88
Serum iPTH (pg/mL)	29.4 ± 16.3	33.2 ± 21.6	39.8 ± 18.7	<0.001
	11.6 ± 8.4	10.9 ± 8.8	9.8 ± 6.0	0.08
Serum 25(OH)D (ng/mL)				

iPTH: intact parathyroid hormone, corrected calcium (mg/dL) = measured total calcium (mg/dL) + 0.8 (4.4- serum albumin (g/dL), where 4.4 represents the average albumin level; Analysis is based on XtGee: generalized estimating equation; *P value less than 0.05, was considered statistically significant.

### Correlation of serum vitamin D levels with iPTH and serum calcium (total, and ionized) in MDR-TB, PTB cat I and healthy controls

**Figure 1.**
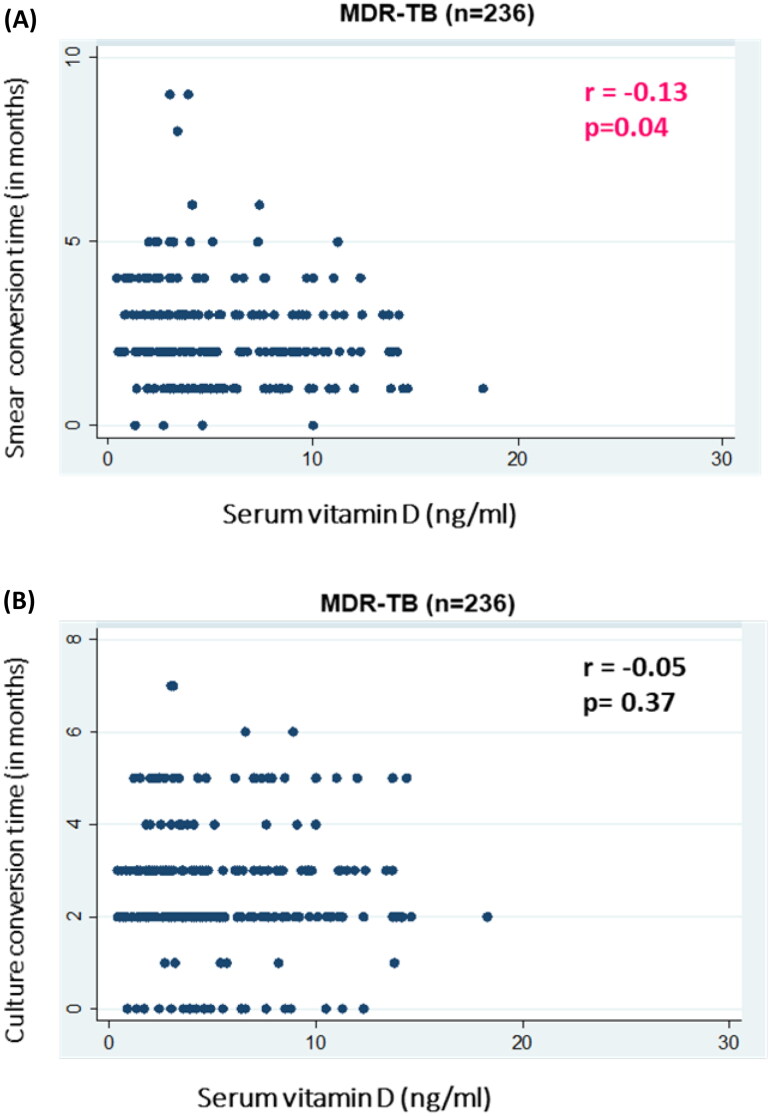
Association of serum vitamin D levels with response to treatment [sputum AFB smear (A) and culture (B)] in patients with MDR-TB. (A) Scatter plot is shown between serum vitamin D levels (ng/ml) on the X-axis and sputum smear conversion time (in months) on the Y-axis with correlation coefficient (r) and significance of correlation (p). (B) (B) Scatter plot of serum vitamin D levels (ng/ml) on the X-axis and sputum culture conversion time (in months) on the Y-axis with correlation coefficient (r) and significance of correlation (p).

**Figure 2.**
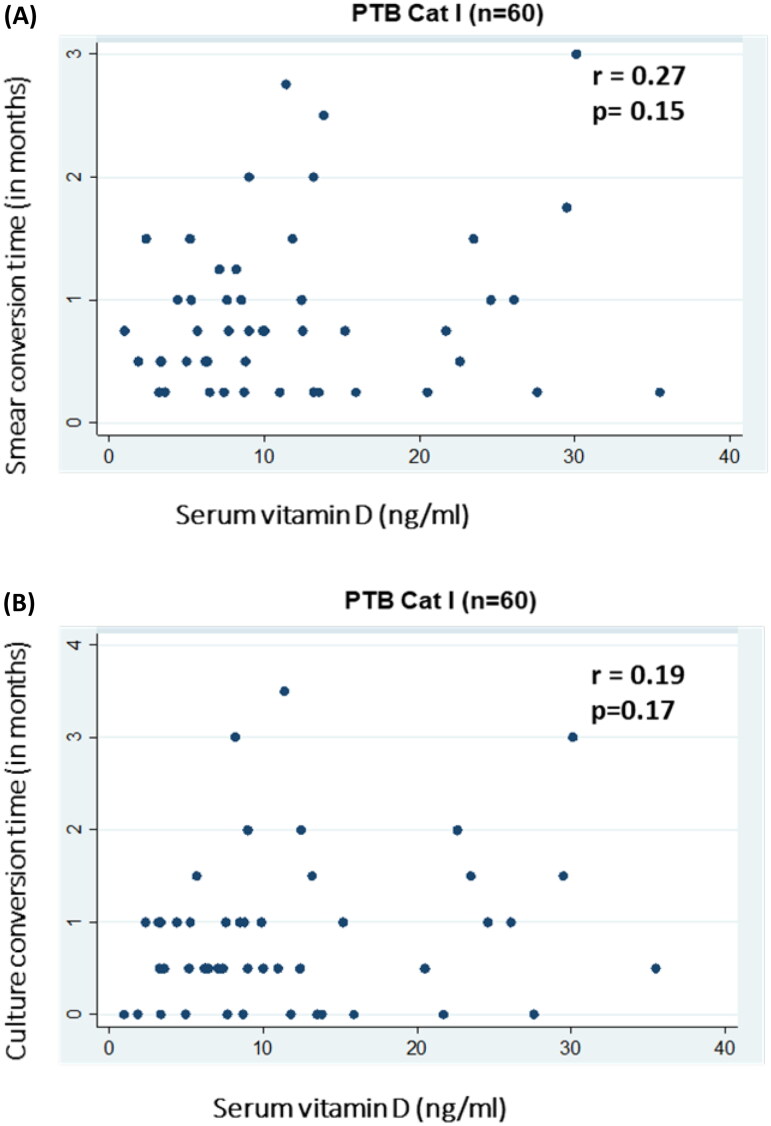
Association of serum vitamin D levels with response to treatment [sputum AFB smear (A) and culture (B)] in patients with PTB cat I. (A) Scatter plot of serum vitamin D levels (ng/ml) on the X-axis and sputum smear conversion time (in months) on the Y-axis with correlation coefficient (r) and significance of correlation (p). (B) Scatter plot of serum vitamin D levels (ng/ml) on the X-axis and sputum culture conversion time (in months) on the Y-axis with correlation coefficient (r) and significance of correlation (p).

In MDR-TB, smear conversion time was significantly negatively correlated with serum vitamin D levels [(Spearman’s rho coefficient −0.13; *p* = 0.04) (Figure 1A)]. However, a non-significant correlation was found in PTB Cat I patients [(*r* = 0.27; *p* = 0.15) (Figure 2A)]. Culture conversion time for both MDR-TB [(r=-0.05; *p* = 0.37) (Figure 1B)] and PTB Cat I [(*r* = 0.19; *p* = 0.17)] showed no statistically significant relationship with serum vitamin D levels (Figure 2B).

**Figure 3.**
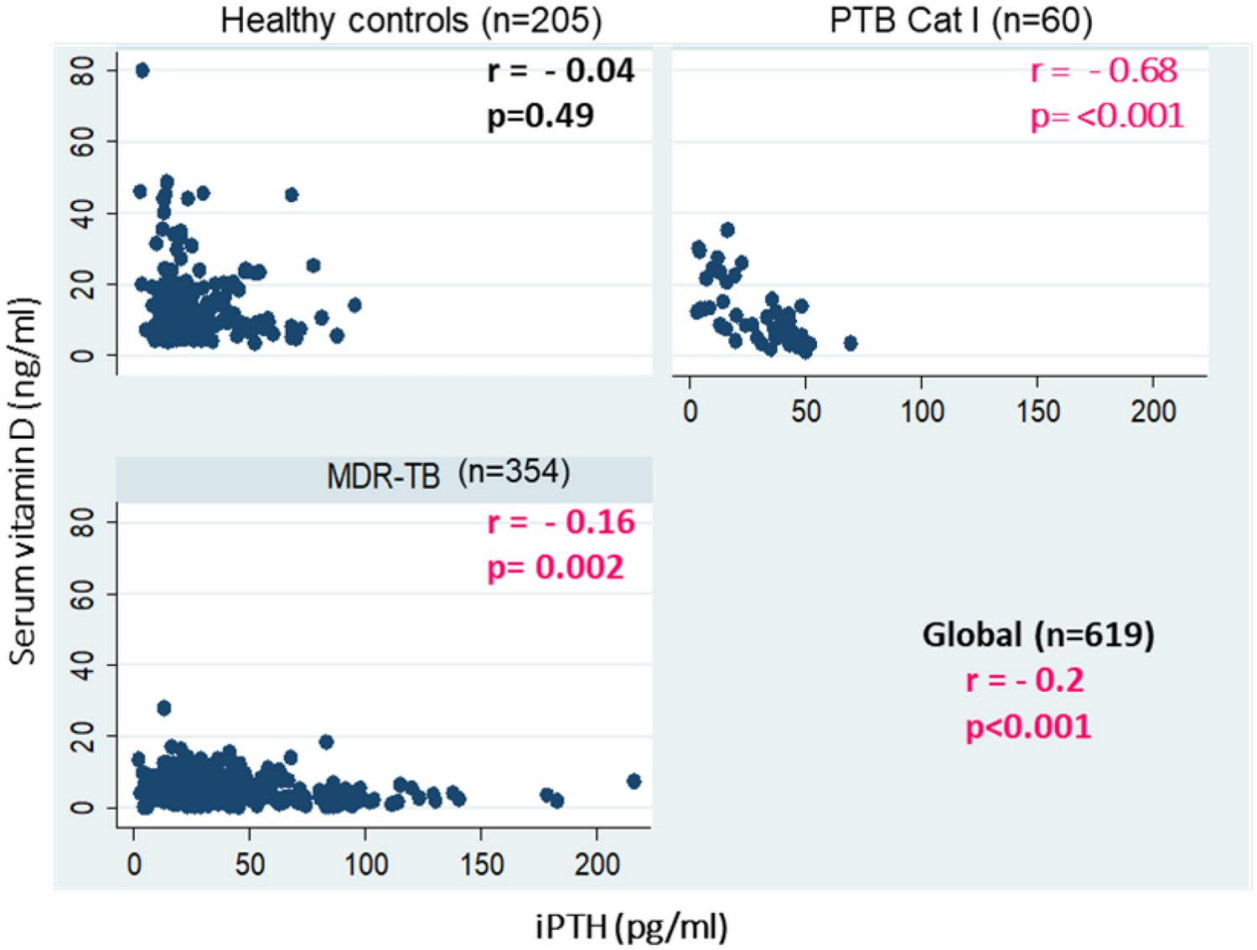
Correlation between serum vitamin D and iPTH levels in MDR-TB, PTB cat I patients and healthy controls. A scatter plot is shown between serum iPTH levels (pg/ml) on the X-axis and serum vitamin D levels (ng/ml)) on the Y-axis with correlation coefficient (r) and significance of correlation (p).

**Figure 4.**
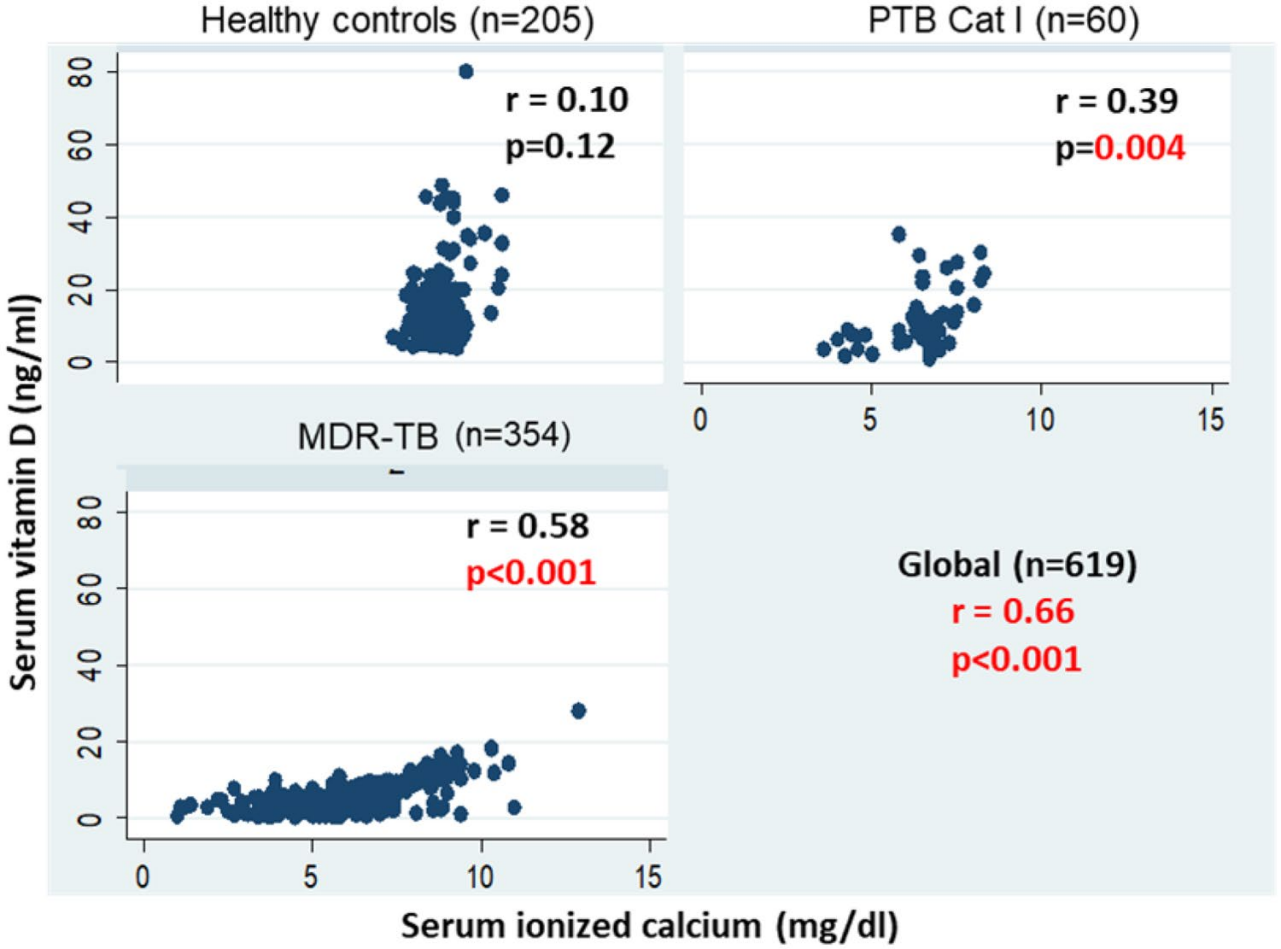
Correlation between serum vitamin D and ionized calcium levels in MDR-TB, PTB cat I patients and healthy controls. A scatter plot is shown between serum ionized calcium (mg/dl) in the X-axis and serum vitamin D levels (ng/ml)) in the Y-axis with correlation coefficient (r) and significance of correlation (p).

**Figure 5.**
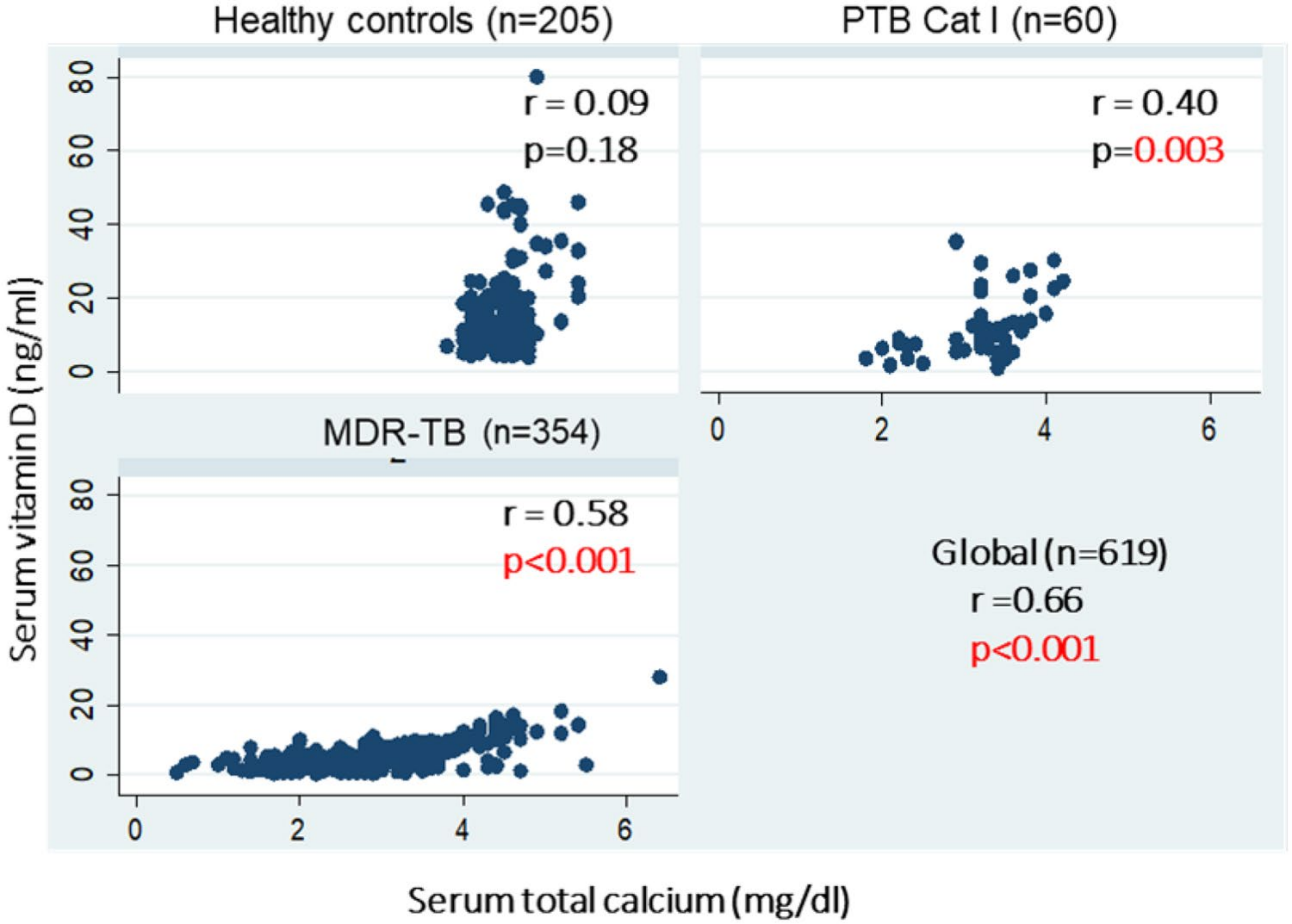
Correlation between serum vitamin D and serum total calcium levels in MDR-TB, PTB cat I patients and healthy controls. Scatter plots are shown between serum total calcium (mg/dl) on the X-axis and serum vitamin D levels (ng/ml)) on the Y-axis with correlation coefficient (r) and significance of correlation (p).

**Figure 6.**
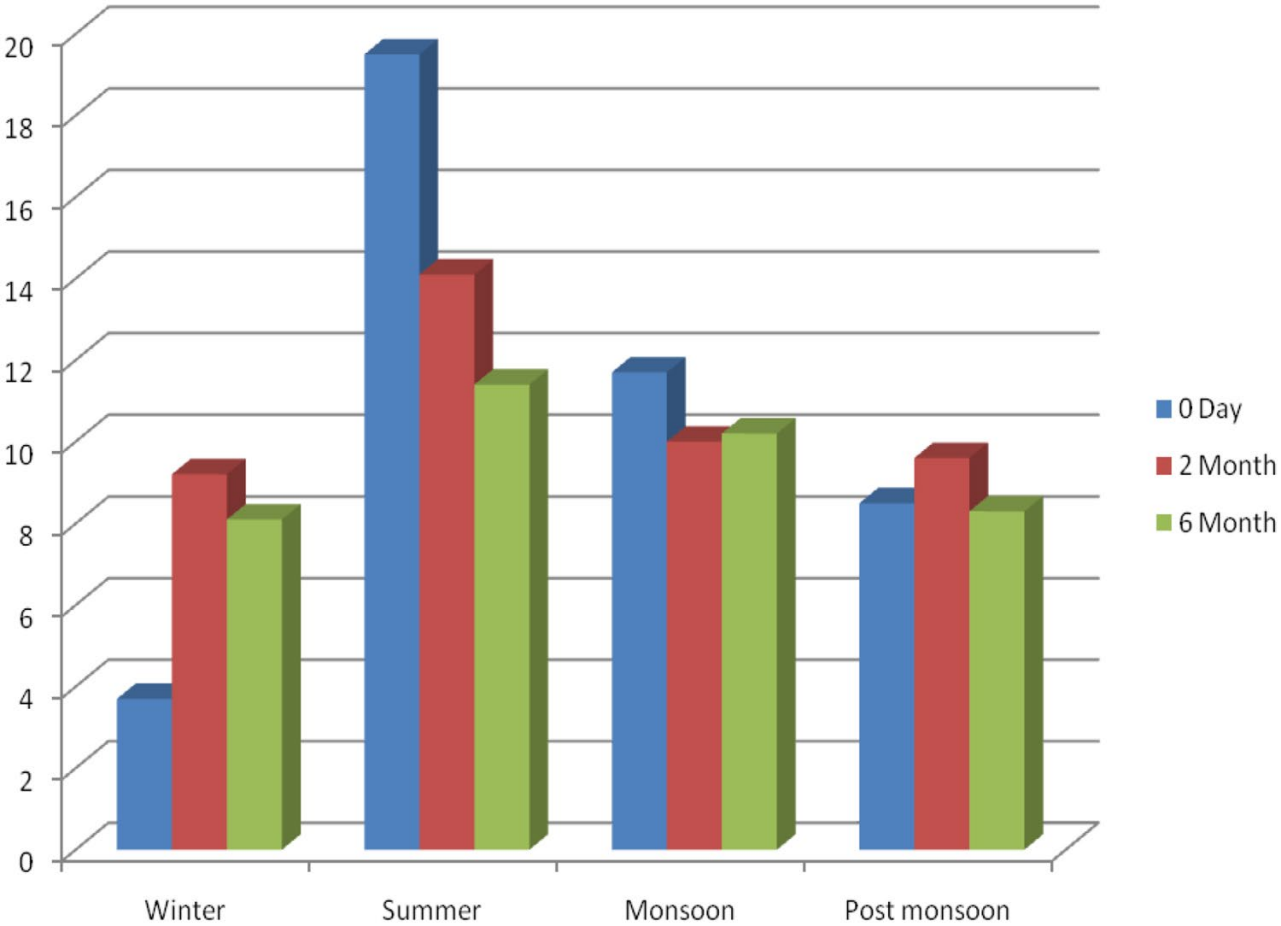
Seasonal variations of serum vitamin D levels levels (ng/ml) in PTB cat I patients at three time points. The bar Graph represents seasonal variations in months (X-axis) of serum vitamin D levels (Y-axis).

An inverse significant correlation between serum vitamin D with iPTH levels was found in MDR-TB patients (r= −0.2, *p* < 0.001) and PTB Cat I patients (r= −0.68, *p* < 0.001), whereas in healthy controls, serum vitamin D levels were not significantly correlated with iPTH levels (r= −0.04, *p* = 0.49). However, globally, an inverse significant correlation between serum vitamin D with iPTH levels was found among the groups (r= −0.2, *p* < 0.001 global) (Figure 3). Likewise, a positive correlation between serum vitamin D levels and ionized calcium levels was found in MDR-TB (*r* = 0.58, *p* < 0.001), PTB Cat I patients (*r* = 39, *p* = 0.004), and healthy controls (*r* = 0.10, *p* = 0.12) (Figure 4). However, globally, a positive significant correlation between serum vitamin D and iPTH levels was found among the groups (*r* = 0.66, *p* < 0.001 global), and a positive correlation between serum vitamin D levels and serum total calcium levels was found in MDR-TB (*r* = 0.58, *p* < 0.001), PTB Cat I patients (*r* = 40, *p* = 0.003), and healthy controls (*r* = 0.09, *p* = 0.18) (Figure 5). The issue of sun exposure during different seasons gives rise to the theory that at different times in the year, the sun intensity and, therefore, exposure will be different. For example, in New Delhi, in the summer, the ground surface receives 4 MED (minimal erythemal dose) of ultraviolet radiation per day, whereas in winter this falls to 1 MED per day [22]; however, the levels for the PTB Cat I group are significantly lower in the winter and post-monsoon seasons than in both summer and monsoon seasons (Figure 6). This exaggerated response to seasonal variation in PTB Cat I compared to non-infected individuals (and those with MDR-TB) has not been found previously [12], and the reason for this is unclear.

## Discussion

Adults are most often affected by pulmonary TB, a communicable bacterial infection of the lungs. It mostly happens as a result of dormant TB bacilli that develop years or even decades after a significant infection, usually when the immune system is weakened [25,30]. When someone coughs, sneezes, spits, or has an active *Mycobacterium tuberculosis* infection, it can spread through the air. The majority of infections in humans begin as asymptomatic latent infections, and about 10% of these eventually become active diseases that, in the absence of treatment, kill over 50% of their victims [31]. Local series and central caseation necrosis are much more marked in post-primary TB than in primary TB, resulting in cavity formation. TB cavities are preferred sites for the growth of *Mycobacterium tuberculosis* because of the accessibility of abundant oxygen and various nutrients. The spread of infection in communes mainly occurs from post-primary (cavitary) TB [30]. Various factors are coupled with an increased threat of infection and, consequently, the development of disease. Pulmonary TB is marked by the formation of granulomas in infected lung tissues and cell-mediated hypersensitivity, which also causes inflammation and fibrocavitary destruction in the lungs, produces chronic respiratory symptoms, and degrades the quality of life [31]. In PTB cat I treatment, an intensive phase with isoniazid (INH), rifampicin, pyrazinamide, and ethambutol for 2 months and a continuation phase comprising the concomitant use of INH and rifampicin for another 4 months was administered [32]. Culture-negative pulmonary TB (PTB) in patients without HIV co-infection is likely an early disease state. If left untreated, it can lead to culture-positive ­disease [33].

A major finding of the present study is patients with MDRTB had substantially lower serum 25(OH)D levels than PTB Cat I patients, who in turn had lower serum vitamin D levels than healthy controls. It has been proposed recently, though there is still uncertainty, that the evidence points to low vitamin D levels as a predisposing factor for tuberculosis rather than TB itself depleting the vitamin D [26]. Though the exact mechanism is unknown, it is plausible that a vitamin D deficiency could predispose treatment-naive PTB Cat I patients to developing MDR-TB. But it’s commonly known that ATT can deplete vitamin D on its own [26]. Transmission is most efficient in poorly ventilated and swarming environments. Droplets become diluted once they enter the peripheral atmosphere, and *Mycobacterium tuberculosis* is rapidly destroyed by ultraviolet radiation. As the Indian TB program stipulates, patients are not routinely tested for drug resistance unless they fail standard ATT, so all the MDR-TB recruited patients had previous partial or full courses of ATT. This may have contributed to the observed lower serum levels in this group, as well as the loss of appetite induced by ATT drugs, leading to reduced macro- and micronutrient intake.

In addition, reinfection is a significant factor in TB spread of TB within highly prevalent populations, such as India [2]. It is also possible that those who are vitamin D-deficient are more likely to be infected and then reinfected with TB. Therefore, they may be more likely to pick up various strains, one of which may be MDR, that is, if the first one is fully susceptible, the next may be resistant. It must be emphasized that the mean 25(OH)D levels for each group were indicative of hypovitaminosis D, which is widely described in otherwise healthy Indian groups [22,34–37]. In light of this, interpretation must be of the impact of the degree of deficiency, as opposed to the simple presence of deficiency, which is more complicated. This means that there is no obvious cutoff or threshold level, and it must be seen as a spectrum. One study from Greenland suggested that both high and low serum vitamin D concentrations predispose patients to active TB [38], which supports our findings. In the present study, during the treatment of MDR-TB, most patients underwent culture conversion within 2 months. The results of chest radiography and sputum culture can help identify patients who might take longer to achieve conversion and, therefore, have a poor treatment outcome. According to one study, the majority of patients achieved sputum culture conversion within three months. Another study found that the median time to culture conversion was two months [39,40] which further supports the facts and objective of the present study. The sputum culture status is a reliable and useful indicator of a patient’s infectiousness [41]. A normal sputum culture can take 1–8 weeks to provide results. Few studies with larger cohorts of non-MDR-TB and healthy controls [42] and [26] on serum vitamin D levels have been conducted in terms of the number of PTB cat I patients [26]. Ionized calcium and intact PTH levels were performed on all subjects to rule out these as confounding factors, and a significantly positive and negative correlation with serum vitamin D levels was found, respectively, which was as expected in patients without disorders affecting calcium homeostasis

In most cases, anti-tuberculosis treatment was administered to MDR-TB patients within a year of their diagnosis. The known effect of anti-tuberculosis medications on vitamin D levels suggests that this could be a confounding factor [43–47]. However, it would be interesting to know whether patients with reduced vitamin D levels experience a higher risk of severe illness due to re-infection with multi-drug resistant strains. This makes sense because it appears more likely than not that a vitamin D deficiency causes a person to become susceptible to TB [43,46,48]. However, 60,000 IU of oral vitamin D will be advised based on studies [19,20]. High serum vitamin D levels were linked to improved lung function and decreased airway inflammation. These findings imply that vitamin D deficiency would confer greater protection for overweight people. Despite conflicting research, vitamin D supplementation may help increase resistance to respiratory infections in general, especially when taken regularly because of its immunomodulatory properties [49]. Research indicates that administering 50,000 IU of vitamin D to asthma patients who have low levels of serum vitamin D (less than 20 ng/mL) enhances their steroid response by upregulating the expression of GR-α, the glucocorticoid receptor, and lowering blood levels of IL-17F and IL-4, two cytokines linked to asthma [50]. According to a different study by Hornsby et al. infants whose moms took 4400 IU/d of vitamin D3 had improved innate immune fitness [51]. The addition of oral phenylbutyrate and vitamin D3 adjunctive therapy (PBA + vitD3 or vitD3 or PBA) to standard short-course therapy has been shown to have positive effects on clinical recovery and may one day be used in host-directed therapy for tuberculosis. This kind of host-directed therapy may be used to combat respiratory infections by boosting innate immunity, increasing antimicrobial activity, and modifying immune responses [52].

The study’s strength is that all parameters were measured in patients with MDR-TB, PTB Cat I patients, and healthy controls. This made it possible to compare patients with TB who did not have baseline MDRTB and patients who did not have active TB in two dimensions.

The comprehensive biochemical profile enabled a detailed comparison of the groups to identify the potential reasons for the observed differences. In comparison to earlier research, the sample size was comparatively large, particularly when it came to MDR-TB. The small number of PTB Cat I patients who underwent serum biochemical analysis in comparison to the other two groups was one of the study’s limitations. Furthermore, because the study was cross-sectional, it is challenging to draw definitive conclusions about the directionality of any association. Furthermore, the measurement of serum vitamin D levels did not follow a strict schedule for the time of year, which can introduce uncertainty due to seasonal variations in sunlight intensity in non-equatorial regions. The period of follow-up is another limitation. Our data may represent the true response to treatment in Cat I PTB compared to MDR-TB because the MDR-TB were only followed up until sputum culture conversion or hospital discharge.

However, it is challenging to account for confounding variables in the Indian population due to its known relatively heterogeneous genetic and behavioral variation, including food and sun exposure [12,46] which may need more research in order to assess the current findings. The current study supports the previous finding that vitamin D did not prevent the time it took for sputum smear and culture conversion, but it could speed up the process. Patients who did not have negative sputum cultures within three months had worse treatment outcomes than those who converted within that same time frame. In addition to vitamin D-fortified foods that were recommended for all patients and the healthy population who are deficient and likely to get infections in the near future, we would like to suggest vitamin D supplementation clinical trials in Indian populations based on the available data. In conclusion, our data suggest that patients with MDR-TB who showed persistent sputum positivity had a worse prognosis than those who showed sputum bacteriology conversion. Two months following the start of treatment, sputum smear conversions may be a useful indicator of how well an MDR-TB patient will respond to treatment.

## Data Availability

Upon reasonable request, the corresponding author, Dr. Jaishriram Rathored, will provide the data supporting the study’s conclusions.
